# Poorly soluble cobalt oxide particles trigger genotoxicity via multiple pathways

**DOI:** 10.1186/s12989-016-0118-8

**Published:** 2016-02-03

**Authors:** Chiara Uboldi, Thierry Orsière, Carine Darolles, Valérie Aloin, Virginie Tassistro, Isabelle George, Véronique Malard

**Affiliations:** 1 Institut Méditerranéen de Biodiversité et d’Ecologie marine et continentale (IMBE), Aix Marseille Université, CNRS, IRD, Avignon Université, Equipe Biogénotoxicologie, Santé Humaine et Environnement, Faculté de Médecine Timone, Marseille, France; 2 CEA, DSV, Institute of Environmental Biology and Biotechnology (IBEB), Perturbed Systems Biochemistry Laboratory (LBSP), Bagnols-sur-Cèze, France; 3 CEA, DSV, Institute of Biology and Technology Saclay (Ibitec-s), Molecular Labeling and Bio-organic Chemistry Unit (SCBM), Gif sur Yvette, France; 4 CEA, DSV, Institute of Environmental Biology and Biotechnology (IBEB), IBEB, Laboratoire des Interactions Protéine Métal, Saint-Paul-Lez-Durance, France

**Keywords:** Cobalt oxide nanoparticles, Human lung cells, Cytotoxicity, Genotoxicity, micronuclei, Comet assay, Foci formation, Oxidative stress

## Abstract

**Background:**

Poorly soluble cobalt (II, III) oxide particles (Co_3_O_4_P) are believed to induce in vitro cytotoxic effects via a Trojan-horse mechanism. Once internalized into lysosomal and acidic intracellular compartments, Co_3_O_4_P slowly release a low amount of cobalt ions (Co^2+^) that impair the viability of in vitro cultures.

In this study, we focused on the genotoxic potential of Co_3_O_4_P by performing a comprehensive investigation of the DNA damage exerted in BEAS-2B human bronchial epithelial cells.

**Results:**

Our results demonstrate that poorly soluble Co_3_O_4_P enhanced the formation of micronuclei in binucleated cells. Moreover, by comet assay we showed that Co_3_O_4_P induced primary and oxidative DNA damage, and by scoring the formation of γ-H2Ax foci, we demonstrated that Co_3_O_4_P also generated double DNA strand breaks.

**Conclusions:**

By comparing the effects exerted by poorly soluble Co_3_O_4_P with those obtained in the presence of soluble cobalt chloride (CoCl_2_), we demonstrated that the genotoxic effects of Co_3_O_4_P are not simply due to the released Co^2+^ but are induced by the particles themselves, as genotoxicity is observed at very low Co_3_O_4_P concentrations.

## Background

The industrial application of cobalt nanoparticles ranges from supercapacitors [1, 2] and pseudocapacitors [3, 4] to sensors [5, 6], while in biomedicine they are mainly employed in magnetic resonance imaging [7] and as nonviral DNA carriers in gene therapy [8–10]. Although the use of cobalt particles has improved many industrial and biomedical applications, their biocompatibility and permanence in tissues and cells remains an open issue.

Human occupational exposure to cobalt particles can be accidental [11, 12] or chronic, and the main route of exposure is inhalation during the production of the particles themselves or of the nanobased products. When dispersed in aqueous solutions, cobalt nanoparticles undergo leaching and release cobalt ions (Co^2+^). This peculiarity has been shown to depend on their chemical form: metallic cobalt and cobalt (II) oxide particles (CoOP) are significantly more soluble than the cobalt (II, III) oxide, Co_3_O_4_P [13, 14]. Nevertheless, while Papis et al. showed that the dissolution of nanosized Co_3_O_4_P in cell culture medium is negligible and not able to reach effective concentrations [15]. In a previous study Sabbioni and colleagues reported a significant Co^2+^ release that resulted, within 72 h, from the dissolution of half of the cobalt metallic particles dispersed in the biological medium [16]. Interestingly, Ortega and coauthors showed that Co_3_O_4_P display a very low solubility at neutral pH in culture medium, but in an acidic environment, as can be found in lysosomes, there is slight particle solubilization leading to a low Co^2+^ release [17]. The role of Co^2+^ in the overall toxicity of Co_3_O_4_P is not yet fully understood, and it is still unclear whether the toxic potential of Co_3_O_4_P is intrinsic or due to ionic release in solution. Compared to cobalt metal micro- and nanoparticles, Co^2+^ derived from soluble cobalt chloride (CoCl_2_) induced a less severe cytotoxicity and did not exert in vitro morphological neoplastic transformation in immortalized mouse fibroblasts [18]. By contrast, in human bladder-, hepatic- and lung-derived cells, CoCl_2_ was significantly more cytotoxic than Co_3_O_4_P [15] and CoOP [19]. Gault et al. showed that CoCl_2_ exerted DNA damage through reactive oxygen species (ROS) production in human keratinocytes [20], whereas Kühnel and coauthors reported that the genotoxic effects of CoCl_2_ were not linked to oxidative stress [21]. Furthermore, CoCl_2_ and cobalt metal nanoparticles were shown to induce distinct effects in mouse fibroblasts in vitro: Co nanoparticles displayed a higher cytotoxicity at short exposure times (2–24 h), and induced genotoxicity and neoplastic transformation, whereas CoCl_2_ was more efficient in the induction of primary DNA damage [22]. Additionally, besides the genotoxic potential of CoCl_2_, cobalt ions were shown to induce epigenetic changes and histone modifications in bronchial and alveolar cells [23]. Moreover, the presence of Co^2+^ derived from poorly soluble Co_3_O_4_P intracellular solubilization was demonstrated to trigger cytotoxicity in human bronchial cells through a Trojan-horse-type mechanism [17], and the same effect was observed in six different cell lines representing lung, liver, kidney, intestine, and the immune system exposed to cobalt metal nanoparticles [24].

While the genotoxic effects exerted by cobalt have been investigated mainly by using the highly soluble cobalt metal or CoOP, there is a lack of information on insoluble forms. In this study, we focused on the effects of poorly soluble submicronic cobalt (II, III) oxide particles (Co_3_O_4_P) that can be inhaled in cases of accidental human exposure [12]. Furthermore, since inhalation is the main route of exposure, we investigated the potential genotoxicity induced by poorly soluble Co_3_O_4_P in BEAS-2B human-derived bronchial epithelial cells. This cell line represents a useful in vitro model for lung epithelium [25], exhibiting the highest homology in gene expression pattern with primary nontumor cells and the lowest number of dysregulated genes compared with in vivo samples [26]. In addition, BEAS-2B are a good model for toxicity studies and they have already been used extensively to assess the toxic potential of particulate or soluble cobalt for which the route of human exposure could be inhalation [17, 27–30].

After characterizing the morphology and mean diameter size of Co_3_O_4_P by, respectively, transmission electron microscopy (TEM) and dynamic light scattering (DLS), we investigated cytotoxicity by quantifying adenosine triphosphate (ATP) and the metabolic activity of BEAS-2B cells. The cytome version of the cytokinesis-block micronucleus assay (CBMN-cyt), performed conformingly to [31, 32], allowed us not just to study the genotoxicity of Co_3_O_4_P by scoring the frequency of chromosome breakage and/or loss, but also to evaluate their cytostatic and cytotoxic effects via the proliferation index and the apoptotic index. Finally, DNA single strand breaks (SSB) and double strand breaks (DSB) were assayed by comet assay and by detecting the phosphorylation of the histone, H2Ax, on serine 139, respectively.

The effects induced on BEAS-2B cells by poorly soluble Co_3_O_4_P were compared with CoCl_2_ by exposing the cells to equal concentrations of cobalt, allowing us to discriminate the respective contributions by the particles themselves and by the ions released into the cell culture medium.

## Results

### Co_3_O_4_P characterization in culture medium

**Fig. 1:**
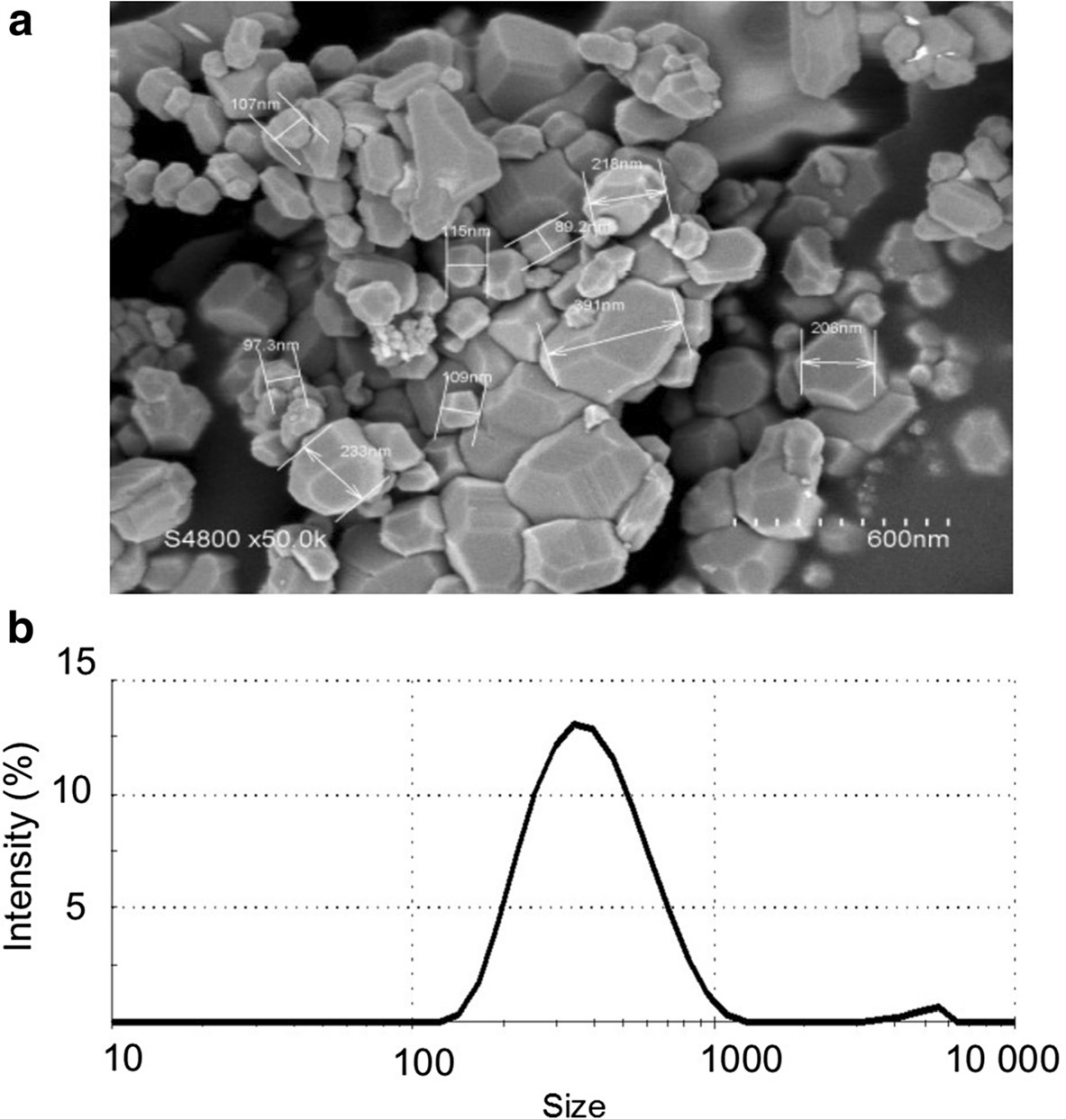
Morphometric analysis and particle size determination of Co_3_O_4_P. Co_3_O_4_P solutions resulted polydispersed and with multiple morphologies, as shown by SEM (**a**). The dispersion of particles in culture medium and after 15 min sonication was analysed by DLS (**b**). The mean intensity ± standard deviation (SD) corresponded to 397.3 ± 175.4 nm, and PDI = 0.21 ± 0.03 (*n* = 6). Both SEM and DLS showed that Co_3_O_4_P solutions were heterogeneous and composed of particles whose size ranged significantly

A detailed description of the physicochemical properties of Co_3_O_4_P has already been presented in previous reports [17, 29]. Scanning electron micrographs (Fig. 1a) showed that Co_3_O_4_P were mainly aggregated and exhibited a polyhedral structure with heterogeneous sizes in the range 100 to 400 nm. Co_3_O_4_P size distribution (Fig. 1b) was further determined by DLS after resuspension of the particle stock in culture medium and sonication. The main intensity peak (98.9 % intensity) corresponded to a mean size diameter of 397.3 ± 175.4 nm, and the polydispersity index was 0.21 ± 0.03 (*n* = 6). Nevertheless, the intensity graph confirmed the heterogeneity of Co_3_O_4_P observed by SEM, since DLS revealed the presence of particles in the range 100 nm–1000 nm.

These differences in the results observed by SEM and DLS are due to the inability of DLS to properly analyse multi-dispersed particles suspensions [33]. This incapability is caused by a non-linear variation of light scattering as a function of size, which increases with the sixth power of their radius and that, consequently, masks the presence of the smaller particles [34].

### Cytotoxicity of cobalt: poorly soluble particles versus cobalt chloride

**Fig. 2:**
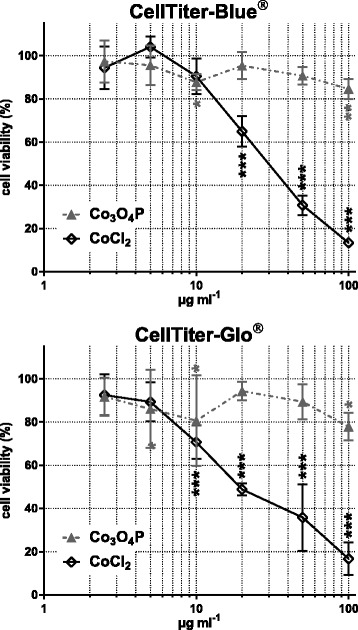
Co_3_O_4_P do not exert cytotoxicity in BEAS-2B cells. CellTiter-Blue® and CellTiTer-Glo® showed that Co_3_O_4_P induced a slight and not statistically significant cytotoxicity in BEAS-2B cells. Either analyzing the mitochondrial activity (CellTiter-Blue®) or the ATP content (CellTiter-Glo®) after 24 h of exposure, the cellular viability was reduced by only about 20 % at the highest concentration tested (100 μg mL^-1^ cobalt). Differently, the cytotoxicity of CoCl_2_ was dose related and at 100 μg mL^-1^ the viability of BEAS-2B was reduced by about 80–85 %. IC_50_ CellTiter-Blue®: 31.30 ± 3.07 μg mL^-1^ cobalt; IC_50_ CellTiter-Glo®: 24.04 ± 3.75 μg mL^-1^ cobalt. Data are presented as mean % ± SEM of two independent experiments in triplicate. Statistical significance was evaluated by one-way ANOVA with Holm-Sidak post-hoc test: *p < 0.05; **p < 0.01; ***p < 0.001

The cytotoxicity of Co_3_O_4_P and CoCl_2_ was evaluated in BEAS-2B cells after 24 h exposure to increasing cobalt concentrations (0 to 100 μg mL^-1^ cobalt). As shown in Fig. 2, no to slight toxicity was observed after exposure to Co_3_O_4_P: the metabolic activity (CellTiter-Blue®) of BEAS-2B cells was impaired by 12.3 ± 1.5 % at 10 μg mL^-1^ (*p* < 0.05) and by 15.4 ± 1.8 % at 100 μg mL^-1^ (*p* < 0.01) whereas, compared with the untreated control, the ATP content (CellTiter-Glo®) was slightly reduced (*p* < 0.05) by 19.4 ± 8.6 % at 10 μg mL^-1^ and by 22.2 ± 2.6 % at 100 μg mL^-1^. The latex beads, LB-3, were not cytotoxic at up to 100 μg mL^-1^ (data not shown), confirming the data reported by Ortega and coauthors [17].

By contrast, CoCl_2_ exerted a severe decrease up to about 85 % in the metabolic activity and in the ATP content in BEAS-2B cells, as shown by CellTiter-Blue® and CellTiter-Glo®. The effect was dose dependent and highly statistically significant (*p* < 0.001) compared with the untreated control (100 % cell viability). Additionally, it was possible to calculate the CoCl_2_ IC_50_, which corresponded to 31.3 ± 3.1 and 24.0 ± 3.8 μg mL^-1^ in the case of CellTiter-Blue® and CellTiter-Glo®, respectively.

Overall, our results show that the cytotoxicity of poorly soluble Co_3_O_4_P is significantly lower than that induced by soluble cobalt chloride. Moreover, our data confirm the results obtained by Darolles et al. [29], who reported no cytotoxic effects in BEAS-2B cells following exposure to Co_3_O_4_P (IC_50_ = 1000 μg mL^-1^ cobalt), and by Bresson and coauthors [28], where, using CellTiter-Glo® assay, the IC_50_ observed for CoCl_2_ corresponded to 20 μg mL^-1^ cobalt.

### Cytostasis and apoptosis induced by poorly soluble Co_3_O_4_P and CoCl_2_

The cytostatic effects exerted by Co_3_O_4_P and CoCl_2_ were evaluated by performing the CBMN assay and by determining the cytokinesis-block proliferation index (CBPI). As shown in Table 1, Co_3_O_4_P in the range 1.25 to 100 μg mL^-1^ cobalt did not induce any statistically significant variation of CBPI compared with the untreated control cells (C neg). Analogously, LB-3 was not cytostatic to BEAS-2B cells. CoCl_2_, by contrast, significantly affected (*p* < 0.05) the cellular proliferation at the two highest concentrations tested, 10 and 20 μg mL^-1^ cobalt, and the impairment of CBPI was equal to or more severe than the positive control (0.1 μg mL^-1^ MMC). CBPI observations were confirmed by calculating the % cytostasis: CoCl_2_ was cytostatic at concentrations ≥ 5 μg mL^-1^ cobalt, and the observed cytostasic effect was comparable to the positive control, MMC; Co_3_O_4_P, in contrast, induced a slight but not statistically significant cytostasis at 10–20 μg mL^-1^, whereas at 100 μg mL^-1^ cobalt, the proliferation of BEAS-2B cells was significantly impaired (*p* < 0.01).

**Table 1:** Cytostasis and cytotoxicity in BEAS-2B cells exposed to Co_3_O_4_P and CoCl_2_

μg mL^-1^		CBPI	% cytostasis	Apoptotic index
C neg		1.61 ± 0.05	0.00 ± 0.00	0.25 ± 0.06
C pos		1.34 ± 0.08*	44.01 ± 4.04***	0.99 ± 0.06***
LB-3		1.64 ± 0.01	0.00 ± 2.43	0.17 ± 0.07
Co_3_O_4_P	1.25	1.62 ± 0.02	0.00 ± 2.57	0.21 ± 0.06
	2.50	1.63 ± 0.02	0.00 ± 2.57	0.34 ± 0.16
	5	1.59 ± 0.06	2.84 ± 3.67	1.61 ± 0.06***
	10	1.48 ± 0.11	20.96 ± 5.57	0.57 ± 0.11
	20	1.48 ± 0.12	20.96 ± 6.02	0.60 ± 0.13
	100	1.39 ± 0.10	36.68 ± 6.49**	0.62 ± 0.02*
CoCl_2_	1.25	1.62 ± 0.01	0.00 ± 2.43	0.06 ± 0.06
	2.50	1.55 ± 0.03	9.43 ± 2.58	0.39 ± 0.15
	5	1.49 ± 0.01	19.34 ± 1.97**	1.02 ± 0.06***
	10	1.34 ± 0.05*	44.01 ± 2.73***	0.60 ± 0.07*
	20	1.20 ± 0.00**	67.07 ± 0.78***	0.10 ± 0.06

The cytostatic effects induced in BEAS-2B after 24 h exposure to Co_3_O_4_P and CoCl_2_ were evaluated by the cytokinesis-block proliferation index (CBPI). Compared with the C neg, only CoCl_2_ at 10–20 μg mL^-1^ cobalt induced a slightly significant reduction of CBPI. By contrast, Co_3_O_4_P and their control, LB-3, did not exert any cytostatic effect on BEAS-2B cells. The % cytostasis confirmed the toxicity of CoCl_2_, but highlighted the significance of the exposure to the highest Co_3_O_4_P concentration tested (100 μg mL^-1^ cobalt). Differently, the cytotoxicity evaluated by scoring the apoptotic index showed that Co_3_O_4_P and CoCl_2_ exerted significant effects at 5 μg mL^-1^, whereas a mild apoptosis was observed after treatment with Co_3_O_4_P and CoCl_2_ (10 μg mL^-1^ cobalt). The positive control, MMC (0.1 μg mL^-1^), was cytostatic and cytotoxic. Data are expressed as mean value ± SEM of two independent experiments, each in duplicate. Statistically significant differences from the C neg were determined by one-way ANOVA followed by Holm-Sidak method for comparisons between groups: *p < 0.05, **p < 0.01 and ***p < 0.001

The apoptosis induced by Co_3_O_4_P and CoCl_2_, as an indicator of cytotoxicity, was investigated by scoring the apoptotic index by CBMN cytome assay. As shown in Table 1, compared with the negative control, Co_3_O_4_P and CoCl_2_ induced cytotoxicity (*p* < 0.001) in BEAS-2B cells at 5 μg mL^-1^ cobalt, with apoptotic indexes of 1.61 % and 1.02 % compared to 0.25 % (negative control), respectively. Co_3_O_4_P at 100 μg mL^-1^ cobalt and CoCl_2_ at 10 μg mL^-1^ cobalt exerted a slight effect, which corresponded to 0.62 % and 0.60 % apoptotic index (*p* < 0.05). The other concentrations tested, and the control latex bead particles LB-3, did not induce apoptosis and no dose effect was observed. Therefore, we cannot conclude to any significant apoptosis induction by cobalt.

### Chromosome damaging properties of cobalt: poorly soluble particles versus cobalt chloride

**Fig. 3:**
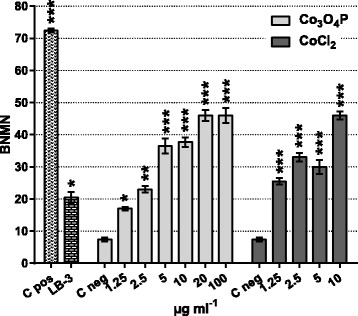
Micronuclei formation in BEAS-2B cells upon exposure to Co_3_O_4_P and CoCl_2_. Compared with C neg (0 μg mL^-1^), Co_3_O_4_P and CoCl_2_ induced statistically significant chromosomal damage or loss with micronuclei formation in BN BEAS-2B cells. BNMN induction was significantly higher following exposure to CoCl_2_ (1.25–2.5–10 μg mL^-1^ cobalt) than Co_3_O_4_P. Mitomycin C (positive clastogenic control; 0.1 μg mL^-1^) and latex beads LB-3 (50 μg mL^-1^) also resulted in a significant induction of MN. Data show the number of BNMN cells ± SEM (two independent experiments, 1000 BN in total). Statistical significance versus C neg was evaluated by chi-square test: *p < 0.05; **p < 0.01; ***p < 0.001

The chromosome damaging potential, evaluated by CBMN assay, showed that Co_3_O_4_P and CoCl_2_ induce micronuclei formation in BEAS-2B cells. As shown in Fig. 3, after 24 h exposure both Co_3_O_4_P and CoCl_2_ exerted a highly statistically significant (*p* < 0.001) and dose-dependent formation of MN in BN cells.

Compared to the C neg, Co_3_O_4_P induced an increase in the number of the BNMN cells that ranged from 2.3-fold at 1.25 μg mL^-1^ cobalt to 6-fold at 100 μg mL^-1^cobalt. The chromosome damaging potential of CoCl_2_ was higher: at 1.25 μg mL^-1^ cobalt, that of BNMN cells was 3.3 times higher than the corresponding negative control, and at 10 μg mL^-1^ the frequency of BNMN was increased by 6-fold. The BNMN cell increase was higher after CoCl_2_ than Co_3_O_4_P treatment at 1.25, 2.5 and 10 μg mL^-1^. Similarly to Co_3_O_4_P, 50 μg mL^-1^ LB-3 induced statistically significant (*p* < 0.05) MN formation, which was enhanced 2.8 times compared with the untreated cells, and consequently the genotoxic potential of LB-3 was comparable to 1.25–2.5 μg mL^-1^ Co_3_O_4_P.

The decision to test CoCl_2_ at a maximum of 10 μg mL^-1^ cobalt was based on the recommendations described by OECD TG487 [35], i.e., to not use test compounds at concentrations inducing more than 55 ± 5 % cytotoxicity.

### Primary and oxidative DNA damage induced by Co_3_O_4_P

To evaluate DNA lesions (single strand breaks), the comet assay was performed, both in its alkaline-conventional (primary damage) and alkaline-modified protocol with the use of restriction enzymes (oxidative damage).

**Fig. 4:**
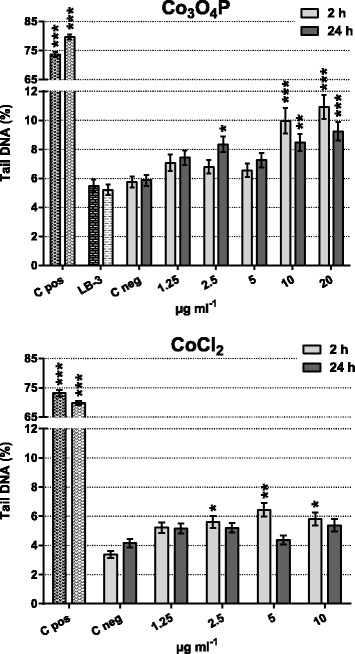
Comet assay showed that poorly soluble Co_3_O_4_P induce primary DNA damage. At 2 h exposure, the primary DNA damage exerted by Co_3_O_4_P was dose dependent and, compared with C neg (0 μg mL^-1^), only 10 and 20 μg mL^-1^ were statistically significant. At 24 h, the effect was not dose dependent, but the measured damage was statistically significant at 2.5, 10 and 20 μg mL^-1^. CoCl_2_, by contrast, did not induce dose-related primary DNA damage at 2 h or at 24 h. A statistically significant increase was observed at 2 h exposure for the three highest concentrations tested whereas no increase was noted at 24 h exposure. Data are presented as mean tail DNA % ± SEM of two independent experiments in duplicate. Statistical significance was evaluated by one-way ANOVA with Holm-Sidak post-hoc test: *p < 0.05, **p < 0.01, ***p < 0.001

As shown in Fig. 4, Co_3_O_4_P induced primary DNA damage in BEAS-2B cells. At short exposures (2 h), the effect observed was dose related although only the two highest conditions tested (10–20 μg mL^-1^) were statistically significant (*p* < 0.001). Moreover, compared with the untreated control, at 10 μg mL^-1^ the increase in DNA damage was 1.7 times higher, while at 20 μg mL^-1^ cobalt there was a 1.9-fold increase. Similarly, after 24 h treatment, Co_3_O_4_P exerted significant DNA strand breaks in BEAS-2B cells at 2.5 μg mL^-1^ (*p* < 0.05) and at 10–20 μg mL^-1^, with an enhanced DNA damage of 1.4, 1.4 and 1.5 times, respectively.

By contrast, CoCl_2_ exerted a milder primary DNA damage (Fig. 4), and at 2 h exposure the DNA strand breaks were slightly more severe than at longer exposures (24 h). In fact, compared with the untreated control, 2 h incubation in the presence of CoCl_2_ induced DNA damage ranging from a 1.5-fold increase at 1.25 μg mL^-1^ to a 1.7-fold increase at 10 μg mL^-1^ cobalt, whereas at 24 h incubation the tail DNA % was enhanced by 1.2-fold. Additionally, statistical significance was observed only after short incubation periods at concentrations superior to 2.5 μg mL^-1^ cobalt. Moreover, comparing the DNA damage exerted by 10 μg mL^-1^ Co_3_O_4_P and CoCl_2_, it was possible to observe that at 2 h exposure the damage, compared with their respective negative control, was of the same intensity (1.7-fold increased tail DNA %); by contrast, at 24 h Co_3_O_4_P was more genotoxic (1.4-fold increase for Co_3_O_4_P vs 1.2-fold increase for CoCl_2_).

To evaluate the oxidative DNA damage caused by cobalt, the enzymes, FPG, which recognizes oxidized pyrimidines, and hOGG1, which is specific for 8-oxoGua, were added to the alkaline comet assay protocol. An analysis of the tail DNA % showed that cobalt particles and cobalt chloride induced oxidative DNA damage both at 2 h and at 24 h exposure (Table 2). Following exposure to Co_3_O_4_P, independently of the length of exposure, FPG detected significant DNA damage at 2.5 μg mL^-1^ and at 10–20 μg mL^-1^ cobalt. Alternatively, the DNA oxidative damage detected by hOGG1 seemed to be more severe at low concentrations (1.25–10 μg mL^-1^cobalt) and at 24 h exposure compared with the results observed at short incubation times. Moreover, as already reported by Smith et al., hOGG1 appeared to recognize oxidative damage with greater specificity than FPG [36].

**Table 2:** Oxidative DNA damage evaluated by alkaline comet assay modified with the enzymes FPG and hOGG1 in BEAS-2B cells: tail DNA %

μg mL^-1^		2 h		24 h	
		FPG	hOGG1	FPG	hOGG1
LB-3		6.22 ± 0.48	5.41 ± 0.46	3.94 ± 0.34	3.33 ± 0.33
C pos		81.89 ± 0.69***	72.25 ± 0.53***	81.78 ± 0.70***	83.01 ± 0.61***
C neg		6.79 ± 0.53	5.15 ± 0.43	6.19 ± 0.48	3.15 ± 0.33
Co_3_O_4_P	1.25	5.60 ± 0.39	15.69 ± 1.49***	4.81 ± 0.35	11.57 ± 0.69***
	2.5	9.59 ± 0.66**	9.01 ± 0.56	8.73 ± 0.55**	12.82 ± 0.82***
	5	6.25 ± 0.52	8.31 ± 0.52	7.39 ± 0.53	9.58 ± 0.77***
	10	11.23 ± 0.76***	13.43 ± 1.78***	10.74 ± 0.64***	6.80 ± 0.61***
	20	12.14 ± 0.76***	13.47 ± 1.46***	10.79 ± 0.73***	4.36 ± 0.39
C pos		82.71 ± 0.42***	76.37 ± 0.61***	73.67 ± 0.64***	74.99 ± 0.77***
C neg		2.98 ± 0.29	2.72 ± 0.26	4.77 ± 0.32	4.31 ± 0.28
CoCl_2_	1.25	4.93 ± 0.33**	4.43 ± 0.34*	5.02 ± 0.36	5.75 ± 0.47
	2.5	6.98 ± 0.47***	5.88 ± 0.46***	4.71 ± 0.38	4.69 ± 0.36
	5	5.01 ± 0.37**	5.73 ± 0.43***	4.93 ± 0.36	6.75 ± 0.42**
	10	7.71 ± 0.48***	5.02 ± 0.41***	5.20 ± 0.37	8.47 ± 0.49***

After 2 h exposure, both FPG and hOGG1 enzymes detected oxidative DNA damage in BEAS-2B cells. Additionally, at the highest concentrations tested, Co_3_O_4_P induced more severe DNA damage than CoCl_2_, but compared with C neg, CoCl_2_ induced statistically significant DNA damage at all the concentrations tested, while Co_3_O_4_P did not. After 24 h, both enzymes showed that oxidative DNA damage occurred in the presence of Co_3_O_4_P, which, additionally, seemed to induce more severe and significant damage than CoCl_2_. LB-3 (50 μg mL^-1^) was used as an internal control for the cobalt particles; 110 μM H_2_O_2_ represented the positive control. Statistical analysis was performed by one-way ANOVA with Holm-Sidak post-hoc test (*p < 0.05; **p < 0.01; ***p < 0.001)

In the case of CoCl_2_, interestingly, the oxidative DNA damage seemed to occur severely after 2 h exposure, at all concentrations tested (1.25–10 μg mL^-1^ cobalt), and independently of the enzyme used. At 24 h exposure, by contrast, only hOGG1 detected statistically significant oxidative DNA damage at 5 and 10 μg mL^-1^, whereas with FPG all the conditions appeared not to exert DNA strand breaks.

LB-3 was not genotoxic at 2 h or 24 h exposure, and the measured tail DNA % was comparable to the C neg (Fig. 4 and Table 2); conversely, the primary (Fig. 4) and oxidative (Table 2) DNA damage induced by the positive control, H_2_O_2_, was statistically significant (*p* < 0.001).

### Generation of γ-H2Ax after cobalt treatment

**Fig. 5:**
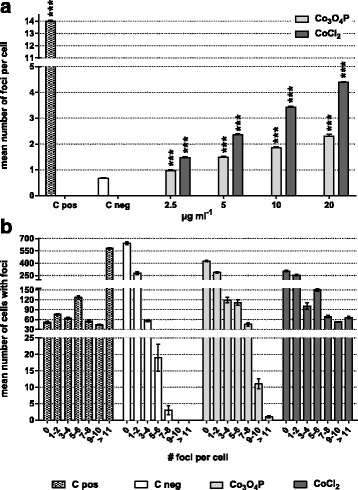
Evaluation of double strand breaks in BEAS-2B cells exposed to cobalt using γ-H2Ax staining. The graphical representation of the phosphorylation of the histone H2Ax as (**a**) mean number of foci per cell indicates that CoCl_2_ is slightly more genotoxic that Co_3_O_4_P. This conclusion is further supported by the mean number of cells that developed foci after exposure to 10 μg mL^-1^ cobalt, (**b**), which clearly shows how CoCl_2_ induced more foci formation than Co_3_O_4_P. C pos (0.10 μg mL^-1^ MMC) was highly significant compared with C neg. Data are presented as (**a**) mean number of foci per cell ± SEM and (**b**) mean number of cells with a given number of foci (two independent experiments in duplicate). Statistical significance was evaluated by one-way ANOVA with Holm-Sidak post-hoc test: ***p < 0.001

By detecting, via immunofluorescence, the phosphorylated histone, H2Ax (γ-H2Ax), we evaluated the DNA double strand breaks (DSB) exerted by 24 h exposure to cobalt, and the results showed that Co_3_O_4_P and CoCl_2_ (10 μg mL^-1^ cobalt) induced a linear γ-H2Ax formation already from 2.5 to 20 μg mL^-1^ cobalt (Fig. 5a). We also observed that Co_3_O_4_P (0.97 ± 0.04 foci per cell at 2.5 μg mL^-1^ up to 2.32 ± 0.07 at 20 μg mL^-1^) induced a less severe formation of γ-H2Ax foci compared with CoCl_2_ (1.47 ± 0.04 at 2.5 μg mL^-1^ up to 4.39 ± 0.02 at 20 μg mL^-1^), while C pos (MMC, 0.1 μg mL^-1^) resulted in a statistically significant higher mean number of γ-H2Ax foci per cell (14.01 ± 0.07) compared with C neg (0.67 ± 0.03).

In the case of BEAS-2B incubated with Co_3_O_4_P (10 μg mL^-1^ cobalt), the mean number of cells with a given number of γ-H2Ax foci in the nuclei (Fig. 5b) decreased and ranged from 425.00 ± 9.74 cells in the group with no foci per cell to 1.00 ± 0.33 cells in the group having ≥ 11 foci per nucleus. A similar trend was observed for C neg, which displayed a mean number of cells with γ-H2Ax foci ranging from 0 (642.00 ± 16.27 cells) to 7–8 (3.00 ± 1.33 cells). In addition, CoCl_2_induced a γ-H2Ax foci formation, but the number of cells with a high number of foci was greater compared with Co_3_O_4_P. In fact, 304.00 ± 10.02 cells did not show foci in their nuclei, whereas 66.00 ± 4.16 of the BEAS-2B cells developed ≥ 11 γ-H2Ax foci. In the positive control, MMC, in contrast, a very high number of cells (582.00 ± 9.00) with ≥ 11 γ-H2Ax foci was scored versus 52.00 ± 3.00 BEAS-2B where no foci were scored.

We also analyzed DNA double strand breakage in the presence of the ROS scavenger, N-acetylcysteine (NAC; 0.5 mM, 2 h pretreatment) (Table 3). We observed that the treatment with NAC induced a highly statistically significant (*p* < 0.001) decrease in the number of cells with γ-H2Ax foci, both in BEAS-2B exposed to Co_3_O_4_P and those exposed to CoCl_2_, as well as in C neg. Additionally, the effect was more pronounced in cells incubated with Co_3_O_4_P compared with CoCl_2_. While γ-H2Ax foci formation decreased by 20- and 55-fold in NAC-pretreated BEAS-2B exposed to 10 and 20 μg ml^-1^ CoCl_2_, respectively, at the same cobalt concentrations the decreases in DNA double strand breakage following incubation with Co_3_O_4_P were, respectively, 58- and 75-fold.

**Table 3:** Histone H2Ax phosphorylation in BEAS-2B cells pre-exposed to NAC

μg mL^-1^		Without NAC pretreatment	With NAC pretreatment	Fold decrease
C neg		0.67 ± 0.06	0.06 ± 0.02^###^	11
Co_3_O_4_P	10	1.74 ± 0.12***	0.03 ± 0.01^###^	58
	20	2.26 ± 0.14***	0.03 ± 0.01^###^	75
CoCl_2_	10	3.37 ± 0.19***	0.17 ± 0.04^###^	20
	20	4.40 ± 0.12***	0.08 ± 0.02^###^	55

The pretreatment of BEAS-2B cells with 0.5 mM NAC exerted a statistically significant reduction of γ-H2Ax foci, independently of the exposure of the cells to Co_3_O_4_P or CoCl_2_. However, the protective effect of NAC in Co_3_O_4_P-incubated cells seems stronger than in BEAS-2B exposed to CoCl_2_. Statistical analysis was performed by one-way ANOVA with Holm-Sidak post-hoc test. Significance versus C neg cells not pretreated with NAC: ***p < 0.001. Significance of NAC pretreated samples versus the corresponding non preincubated ones: ^###^ *p* < 0.001

## Discussion

Exposure by inhalation, either occupational or accidental, to metallic cobalt or cobalt oxide particles has increased recently, in line with an increase in their industrial use. In the case of poorly soluble Co_3_O_4_P, the increased risk following inhalation is enhanced by the fact that, in vivo, Co_3_O_4_P can be retained for periods of time ranging from months to years, thus prolonging their toxic potential [12, 37]. In addition, Co_3_O_4_P trigger toxic effects via a Trojan-horse like mechanism, by which Co_3_O_4_P are able to release cobalt ions (Co^2+^) when they are taken up into acidic intracellular compartments such as endosomes and lysosomes [13, 17, 38, 39]. As Co^2+^ are known to be cytotoxic, genotoxic and potentially carcinogenic to humans [40], and because Co^2+^ do not display a threshold below which they are not toxic, cobalt now appears even more potentially harmful for human health.

The current literature reports extensively on the toxicity exerted by soluble cobalt particles (metallic CoP and CoOP oxides) and by their released ionic fraction. For example, the concentration-related reduction in cell viability observed in A549 and in HaCaT was reported by Horie et al. to depend on the released Co^2+^ rather than on CoOP [41]. Similarly, by MTT assay, Chattopadhyay and coauthors reported that chitosan-modified CoOP induced an impairment of mitochondrial activity in different human cell lines such as T-lymphocyte (Jurkat) or myelogenous leukemia (K562 and KG1a) cells, and that cytotoxicity depended on the pH and the amount of Co^2+^ released [42]. In fact, the fraction of cobalt ions released from chitosan-coated CoOP was greater in acidic conditions than at neutral pH, and this effect was positively related to the cytotoxicity of the particles [42].

By contrast, there is also evidence in the literature that cobalt particles are more toxic than cobalt ions. In HepG2 cells, the concentration- and time-dependent cytotoxicity of poorly soluble Co_3_O_4_P, evaluated by measuring mitochondrial activity and membrane integrity, was more severe following exposure to particles than to CoCl_2_ [43]. Similarly, Ponti et al. showed that metal CoP, whose mean size diameter ranged from 20 to 500 nm and which showed a time-dependent Co^2+^ leakage, at 2 and 24 h of exposure, more severely impaired the colony-forming efficiency of Balb/3 T3 mouse fibroblasts compared with Co ions, and this effect was related to a higher cellular uptake of CoP compared with Co^2+^ [22]. More recently, Sabbioni and coauthors showed that the cytotoxicity of metal CoP in Balb/3 T3 fibroblasts was positively related to concentration and the exposure length, with CoP more toxic than Co^2+^ [18]. However, as CoP were highly soluble (10–20 % solubilization within 4 h incubation in culture medium) and the uptake of cobalt released from the particles was 50–60 times higher than CoCl_2_ [44], and considering cytotoxicity as a function of the intracellular Co content, the authors observed that the ionic form (CoCl_2_) induced higher toxicity than the particles [18].

In our study, conversely, we focused on poorly soluble Co_3_O_4_P, which were previously characterized by Ortega and coauthors [17]. By performing CellTiter-Glo® and CellTiter-Blue® assays, and by analyzing cytostasis via the cytokinesis-block proliferation index, we observed that CoCl_2_ induced a severe cytotoxicity, while Co_3_O_4_P did not trigger any cytotoxicity but moderate cytostasic effect in BEAS-2B cells up to 100 and 20 μg mL^-1^, respectively. In agreement with Bresson et al. [28], who set the IC_50_ at 20 μg mL^-1^, we showed that the CoCl_2_ IC_50_ ranged from 24 to 31 μg mL^-1^ at 24 h exposure. In the same way, our results confirmed the noncytotoxic potential of poorly soluble Co_3_O_4_P that has already been reported by Darolles et al. [29], who obtained an IC_50_ > 1000 μg mL^-1^.

Poorly soluble Co_3_O_4_P are characterized by a quick internalization into the lysosomal compartments of BEAS-2B cells. The exposure of BEAS-2B cells to 50 μg mL^-1^of cobalt particles, that corresponds to IC25, gave a quantity of intracellular cobalt particles of 9500 ± 1650 fg/cell, with 6.7 ± 3.0 fg/cell solubilized cobalt, and exposure of BEAS-2B cells to 170 μg mL^-1^of cobalt particles, that corresponds to IC50, gave a quantity of intracellular cobalt particles of 20 450 ± 23 500 fg/cell, with 48 ± 20 fg/cell solubilized cobalt [17]. It was also shown that IC25 CoCl_2_ (2.9 μg mL^-1^) corresponded to 5.4 ± 0.8 fg/cell of intracellular solubilized cobalt, indicating that 17 times more cobalt particles concentration compared to CoCl_2_ are needed to obtain a comparable intracellular amount of solubilized cobalt [17]). Moreover, Co_3_O_4_P are characterized by a very low and pH-dependent Co^2+^ release (up to 3 days in solution, 0.3 % Co^2+^ leaking at neutral pH and 2 % under acidic conditions) [17]. More importantly, they can be solubilized into intracellular organelles (such as endosomes and lysosomes) thus triggering cytotoxicity via a Trojan-horse-like mechanism, [17].

Current literature reports few information on the genotoxic potential of poorly soluble Co_3_O_4_P [30, 43] (Table 4). Most of the studies have been performed on metal Co particles that are known to be genotoxic but soluble [18, 22, 45–48] (Table 4). We investigated the DNA and chromosome damage potentials of these particles versus CoCl_2_. By performing CBMN assays we observed that both Co_3_O_4_P and CoCl_2_ exerted similar MN formation in BN BEAS-2B cells. It is noteworthy that cobalt induced chromosome damage without impairing neither ATP cellular content nor cellular metabolism capability (Fig. 2). Nevertheless, an increase in cytostasis was observed for CoCl_2_ and Co_3_O_4_P at cobalt concentration ≥ 5 and 10 μg mL^-1^, respectively (Table 1). Moreover, alkaline comet assays showed that, at cobalt concentration inferior to 5 μg mL^-1^, the DNA damage exerted by Co_3_O_4_P and CoCl_2_ was comparable, while at 10 μg mL^-1^, Co_3_O_4_P was more genotoxic than soluble CoCl_2_. Lastly, when we analyzed the extent of phosphorylated histone, H2Ax, we observed that CoCl_2_ induced a higher level of damage than Co_3_O_4_P, either considering the mean number of total foci per cell, or taking into account the number of cells with more than 9 foci. This damage might be linked to oxidative effects on the DNA as is was greatly reduced in the presence of NAC.

**Table 4:** Genotoxicity studies on cobalt particles

Type of cobalt particle and nominal size	Cell model	Assays performed, concentration and exposure length	Results	Ref.
Co metal particles				
CoP (3.4 nm) CoMP (2.2 μm)	Balb/3 T3 mouse fibroblasts, clone A31-1-1	Colony forming efficiency (0.1–100 μM, 4–72 h)	Concentration- and time-dependent cytotoxicity	18
		H_2_DCFDA (1–100 μM, 4 h)	Increased intracellular ROS	
		GSH (1–100 μM, 4 h)	Reduced total GSH content	
		LPO (1–100 μM, 4 h)	Concentration-dependent LPO formation	
		Morphological transformation +/- ascorbic acid (1–20 μM, 72 h)	Induction of type-III foci, significantly decreased in the presence of ascorbic acid	
CoP (20–500 nm)	Balb/3 T3 mouse fibroblasts, clone A31-1-1	Colony forming efficiency (0.1–100 μM, 2–24–72 h)	Concentration-related cytotoxicity	22
		Morphological transformation (1–30 μM, 72 h)	Increase in Type-III foci formation	
		CBMN (1–10 μM, 24 h)	Statistically significant induction of MN	
		Comet assay (1–5 μM, 2 h)	Induction of DNA damage	
CoP (100–500 nm)	Human peripheral blood leukocytes (PBLs)	CBMN (10^-6^-10^-5^ μM, 24 h)	Increase in MN formation	45
		Comet assay (10^-5^-10^-4^ μM, 2 h)	Concentration-dependent DNA damage	
CoP (<50 nm)	Mouse embryonic fibroblasts MEF Ogg1^+/+^ MEF Ogg1^-/-^	Automated cell counting method (0.05–40 μg ml^-1^, 24–48 h)	Cytotoxicity observed at 48 h exposure	46
		Comet assay (0.05–1 μg ml^-1^, 24 h)	No DNA damage	
		FPG-modified comet assay (0.05–1 μg ml^-1^, 24 h)	DNA damage in MEF Ogg1^-/-^ cells	
CoP (20 nm)	Human lung epithelial cells (A549)	Alamar blue (2.5–40 μg ml^-1^, 24 h)	Significant cytotoxicity at > 20 μg ml^-1^	47
		H_2_DCFDA (2.5–15 μg ml^-1^, 12 h)	Concentration-dependent increase in ROS generation	
		8-OHdG +/- NAC pre-treatment (2.5–15 μg ml^-1^, 12–24 h)	Oxidative stress and damage but not when cells are pre-incubated with NAC	
		Comet assay (5–15 μg ml^-1^, 12 h)	Concentration- and time-related increase in DNA damage	
		γ-H2Ax foci (5–15 μg ml^-1^, 12 h)	Pre-incubation of cells with NAC attenuated the DNA damage	
CoP (4 μm)	Human peripheral blood mononucleated cells (PBMC)	CBMN (0.6–6 μg ml^-1^, 15 min)	Statistically significant concentration-dependent increase in MN	48
		Comet assay (0.6–6 μg ml^-1^, 15 min)	No DNA damage	
Co_3_O_4_ particles				
Commercially available Co_3_O_4_P Sigma- Aldrich (22 nm)	Human lung epithelial cells (A549)	LDH and WST-1 (1–40 μg ml^-1^, 0.5–2–24 h)	No cytotoxicity	30
		Comet assay (1–40 μg ml^-1^, 2–24 h)	DNA damage at the highest concentrations (20–40 μg ml^-1^)	
		FPG-modified comet assay (1–40 μg ml^-1^, 2–24 h)	Oxidative DNA damage at the highest tested concentrations (20–40 μg ml^-1^)	
	Human bronchial epithelial cells (BEAS-2B)	LDH (1–40 μg ml^-1^, 0.5–2–24 h)	Dose-related cytotoxicity only at 2 h exposure	
		WST-1 (1–40 μg ml^-1^, 24 h)	Statistically significant viability reduction only at 40 μg ml^-1^	
		Comet assay (1–40 μg ml^-1^, 2–24 h)	Concentration-related DNA damage only at 40 μg ml^-1^	
		FPG-modified comet assay (1–40 μg ml^-1^, 2–24 h)	Oxidative DNA damage	
Commercially available Co_3_O_4_P, Sigma-Aldrich (264 nm by DLS; 22 nm by TEM)	Human hepatocarcinoma (HepG2) cells	LDH and MTT (5–40 μg ml^-1^, 24–48 h)	Concentration- and time-dependent cytotoxicity	43
		GSH/LPO/SOD/catalase (5–10–15 μg ml^-1^, 24–48 h)	Concentration- and time-related depletion of GSH and induction of LPO, SOD, and catalase	
		Caspase-3 (5–10–15 μg ml^-1^, 24–48 h)	Concentration- and time-dependent increase of caspase-3 activity	
		Comet assay	Concentration- and time-dependent DNA damage	

By comparing the in vitro genotoxicity of soluble metal CoP and Co^2+^ on freshly isolated human peripheral leukocytes, Colognato et al. observed a clear concentration-dependent MN frequency after exposure to cobalt ions, whereas CoP showed only minor changes compared with the untreated control [45]. Nevertheless, by comparing the individual blood donors, the frequency of MN was highly variable and the differences observed among the single donors were statistically significant [45]. By contrast, as shown by comet assay, CoP induced concentration-dependent primary DNA damage to freshly isolated human peripheral leukocytes, whereas Co^2+^ did not exert any effect, and to explain these controversial results the authors suggested that the lack of DNA strand break induction by CoCl_2_ might be due to the fact that Co^2+^ are internalized less efficiently than CoP [45].

In Balb/3 T3 mouse fibroblasts, soluble CoP was more severe in inducing MN formation compared with Co^2+^: 1–5–10 μM CoP exerted statistically significant but not dose-related chromosomal aberrations, whereas under the same experimental conditions Co^2+^ did not exert micronucleus formation [22]. Additionally, Ponti and coauthors observed, by comet assay, a statistically significant induction of DNA damage following exposure to CoP and Co^2+^, with the increased DNA damage induced by CoP not dose dependent whereas a dose-dependent effect was observed for Co^2+^ [22].

More recently, poorly soluble Co_3_O_4_P were shown to induce, in HepG2 cells, concentration- and time-dependent primary DNA damage [43]. In BEAS-2B cells, we have observed a similar effect following 2 h and 24 h exposure.

Additionally, Cavallo et al. observed that, in BEAS-2B cells, the primary DNA damage induced by Co_3_O_4_P could be observed only at high concentrations (20–40 μg mL^-1^), while oxidative DNA damage, analyzed by performing a FPG-modified comet assay, was particularly evident at low concentrations (5–10 μg mL^-1^) [30]. These results are only partially confirmed by our observations, by which the incubation of BEAS-2B cells with poorly soluble Co_3_O_4_P enhanced primary DNA strand breaks already at 10 μg mL^-1^ cobalt, whereas at 2 h and 24 h exposure oxidative DNA lesions were significantly induced at much low cobalt concentrations (2.5 μg mL^-1^ cobalt using FPG and 1.25 μg mL^-1^ cobalt using hOGG1).

The genotoxic potential of several types of nanoparticles, including cobalt nanoparticles, has been linked to their ability to induce oxidative stress [49–51]. For example, soluble CoOP exerted significant induction of ROS in human lymphocytes and in mouse peripheral blood mononuclear cells [52], as well as in the leukemic Jurkat, K562 and KG1-a cells [53]. In HepG2, the depletion of GSH and the induction of membrane lipid peroxidation was higher in the presence of poorly soluble Co_3_O_4_P than following exposure to CoCl_2_ [43]. Similarly, Papis and coauthors reported that, at equal cobalt concentrations, insoluble Co_3_O_4_P were more capable of inducing ROS in HepG2 and ECV-304 compared with CoCl_2_ [15]. Moreover, the exposure of primary human aorta (HAECs) and umbilical (HUVECs) endothelial cells to poorly soluble Co_3_O_4_P resulted in a statistically significant ROS increase, associated with lipid peroxidation and GSH scavenger activity [54]. We also investigated the oxidative stress induced by cobalt in BEAS-2B cells and we observed that CoCl_2_, but not Co_3_O_4_P, altered the GSH/GSSG ratio at 24 h exposure (data not shown). The difference observed between our results and the literature might be due to either the different solubility of the particles studied (CoOP and Co_3_O_4_P) or to different exposure times. Indeed, oxidative potential of poorly soluble Co_3_O_4_P has been reported following exposure times ranging between 30 and 60 min [15, 54].

Intracellular uptake and solubilization of cobalt were determined in our previous study, showing that the same amount of intracellular soluble cobalt is found when cells are incubated with 50 μg mL^-1^ of particulate cobalt (Co_3_O_4_P) or 2.9 μg mL^-1^ of soluble cobalt (CoCl_2_) [17]. As, in this study, DNA and chromosome damage were observed at low Co_3_O_4_P concentrations (≥2.5 μg mL^-1^), the genotoxic effects would be induced by the particles themselves and not by the amount of intracellular solubilized cobalt which is, as shown in our previous study [17], very low.

Taking our results together, they suggest that Co_3_O_4_P and CoCl_2_ exert genotoxicity by different mechanisms. Co_3_O_4_P seem to induce more primary single DNA strand breaks (SSB) than free Co-ions that, indeed, exert double DNA strand breakages (DSB) in BEAS-2B cells. The main difference between the damage induced by Co_3_O_4_P and CoCl_2_ seem to be related to the contribution given by oxidative stress. The hypothesis is that Co_3_O_4_P generate free radical species on the cell membrane, inducing lipid peroxidation. Lipid peroxidation products, such as malondialdehyde (MDA) and 4-hydroxy-2-nonenal (4-HNE), are well known to contribute to the formation of interstrand DNA crosslinks and DNA-protein conjugates [55]. These DNA lesions are known to decrease the migration of DNA in the comet assay and are converted in double strand break during mitosis or repaired by recombination.

Co-ions also exerted oxidative stress in BEAS-2B cells but not preferentially on cell membrane. The hypothesis is that CoCl_2_ does not induce the same level of lipid peroxydation products, and thus lower interstrand DNA crosslinks and DNA-protein conjugates are generated. Therefore, free radicals species induce a dose related increase in the modified comet assay. Following CoCl_2_ incubation, the formation of DSB is demonstrated by the scoring of MN, but especially by the high phosphorylation of the histone H2AX. Since γ-H2AX foci were inhibited by pre-treating BEAS-2B cells with the antioxidant NAC, and because the GSH/GSSG ratio decreased only in CoCl_2_ exposed cells, we can assume that, compared to poorly soluble Co_3_O_4_P, differently generated free radicals and oxidative stress occur and cause the genotoxicity of cobalt chloride.

Although comet assay is usually known to be more sensitive to DNA damage than CBMN, in our experiments we have observed a substantially greater degree of DNA damage with the CBMN assay. This discrepancy might be linked to the differences of the two proposed protocols. In fact, while comet assay is performed directly at the end of the exposure period, CBMN requires a further incubation (28 h) following exposure to Co_3_O_4_P and CoCl_2_, during which two different events could have happened: (i) cells might have continued being exposed to particles (particles that cannot be completely removed from culture substrates or cell membrane by simple washing), and (ii) the internalized cobalt particles and soluble cobalt might have continued to exert their genotoxic action, resulting thus in a higher genotoxicity compared to comet assay. This discrepancy could also be partly due to a reduced DNA migration during comet assay following interstrand DNA crosslinks and/or DNA-protein conjugates formation consecutive to lipid peroxidation.

## Conclusions

From the current literature (summarized in Table 4), it was evident that there was a lack of information on Co_3_O_4_ particles, which result particularly important since they are those involved in cases of accidental contamination in the nuclear industry [12]. Therefore, our study represents the first comprehensive genotoxicity study on poorly soluble Co_3_O_4_ particles. Our findings, which raise concern about long-term Co_3_O_4_P exposure in case of accidental inhalation, have shown that Co_3_O_4_P induce in BEAS-2B bronchial cells genotoxic effects that are independent of the amount of intracellular solubilized cobalt. DNA and chromosome damages, in fact, are observed at non cytotoxic concentrations and might be linked to oxidative effects on the DNA. In vivo studies would be needed to fully evaluate the carcinogenic risk associated with exposure to these particles, as well as additional in vitro studies to better evaluate the oxidative potential of cobalt particles and cobalt ions.

## Methods

### Reagents

LHC9 and LHC basal medium, trypsin, PBS and ProLong® Gold antifade reagent with DAPI were purchased from Life Technologies (Saint Aubin, France). Co_3_O_4_ particles (Co_3_O_4_P) were supplied by Merck (Fontenay Sous Bois, France) and, according to the manufacturer’s quality control sheet, their purity was of 98.4 %. CoCl_2_ x 6 H_2_O and Polystyrene Latex Beads (LB-3 in aqueous suspension; 0.3 μm mean diameter size) were purchased from Sigma-Aldrich (Lyon, France). CellTiter-Glo® Luminescence Cell Viability Assay and CellTiter-Blue® Assay were purchased from Promega (Charbonnieres, France). All other reagents were purchased from Sigma-Aldrich.

### Cobalt and latex bead preparations

The cobalt particles used were the very same previously investigated by Ortega et al. [17] and by Darolles et al. [29]. Poorly soluble Co_3_O_4_P were suspended in deionized water to prepare stock solutions with a cobalt concentration of 8 mg mL^-1^; suspensions were then sonicated for 15 min with an Autotune sonicator (Fisher Scientific; Illkirch, France) operated at 750 W, and stored at − 20 °C until use. Before the cells were exposed to Co_3_O_4_P, the stock solutions were sonicated for 15 min in the same conditions as described above, and diluted in culture medium [29]. The cobalt concentration of Co_3_O_4_P stocks was determined by ICP-AES from an external standard calibration curve using three wavelength emission lines (228.616 nm, 237.862 nm and 238.892 nm) with a 5 s integration time. Five replicates for each wavelength were analyzed, and the final concentration considered was the mean value obtained from the three wavelengths. Detailed particle characterization has been described previously [17, 29]. Before each experiment, the dispersion or aggregation/agglomeration of freshly sonicated particles was assessed by dynamic light scattering (DLS) using a Nano ZS ZetaSizer (Malvern; Orsay, France).

LB-3 polystyrene latex beads were used as the negative control for Co_3_O_4_P. Before the addition of LB-3 to culture medium, the solution underwent sonication for 1 min [17].

CoCl_2_ x 6 H_2_O (named CoCl_2_), included in the study to discriminate between the toxic effects exerted by Co_3_O_4_P and their released ions, was prepared at a final cobalt concentration of 10 mg mL^-1^ in deionized water, and did not require any sonication step.

### Cell cultures and exposure to cobalt

The transformed human bronchial epithelial cell line, BEAS-2B, was obtained from the American Type Culture Collection (CRL#9609). Cells were cultured in sterile tissue culture treated flasks or plates precoated using a solution comprising BSA (0.01 mg mL^-1^), human fibronectin (0.01 mg mL^-1^) and collagen (0.03 mg mL^-1^) in LHC basal medium. The cultured cells were maintained in LHC-9 medium under standard cell culture conditions (37 °C in 5 % CO_2_ at 95 % humidity) and passaged, by trypsinization (0.25 % trypsin and 2.6 mM EDTA), at 70–80 % confluence.

For the experiments with cobalt, BEAS-2B cells were exposed for 2 h and/or 24 h to increasing concentrations of poorly soluble Co_3_O_4_P and cobalt chloride solutions so that the concentration of cobalt ranged from 1.25 to 100 μg mL^-1^ in LHC-9 medium. As the treatments were performed in multiwell plates or chambers, the volumes were strictly adjusted to the surface area of the culture supports. Cells were also exposed to LB-3 at the fixed, nontoxic concentration of 50 μg mL^-1^ [17].

### Cytotoxicity assays

The effects of poorly soluble Co_3_O_4_P, CoCl_2_ and LB-3 on the viability of human BEAS-2B cells were evaluated using the CellTiter-Blue® Assay and the CellTiter-Glo® Luminescence Cell Viability Assay.

The CellTiter-Blue® assay is based on the measurement of mitochondrial reductase activity, and in particular of resazurin, a nonfluorescent substrate, which is reduced to the fluorescent product, resorufin, by mitochondrial reductases. To perform this assay, 2 x 10^4^ BEAS-2B cells cm^-2^ were seeded into opaque 96-well plates and grown for 24 h before being exposed to Co_3_O_4_P, CoCl_2_ or LB-3. The assay protocol described by the manufacturer was improved to avoid particle interference during luminescence reading [29]: at the end of the exposure period (24 h), 20 μL CellTiter-Blue® solution were added into each well and incubated (3 h at 37 °C). After centrifugation (900 g, 5 min, RT) to pellet Co_3_O_4_P, 100 μL supernatant from each well was transferred into an empty plate, and fluorescence (excitation at 560 nm and emission at 590 nm) was measured on a plate reader (LumiStar, BMG LABTECH, Champigny s/Marne, France). For each condition, three independent assays were carried out, each performed in duplicate. The fluorescence values were normalized to the untreated control and expressed as percentage of viability.

The cytotoxic potential of poorly soluble Co_3_O_4_P on BEAS-2B cells was further investigated by the CellTiter-Glo® Luminescence Cell Viability Assay, an in vitro test that allows the measurement of the amount of intracellular ATP, which is directly linked to the number of metabolically active cells. Briefly, BEAS-2B were plated at the same density and conditions described for CellTiter-Blue®. To avoid interference between Co_3_O_4_P and the CellTiter-Glo® reagents, at the end of the exposure (24 h) plates were handled as described above. Data were acquired using a luminescence plate reader. For each experimental point, three independent assays were carried out, each performed in duplicate. Values were expressed as percentage of viability following the formula [(mean luminescence for a given sample condition/mean luminescence of unexposed cells) x 100].

### Cytostasis, cytotoxicity and genotoxicity: cytome cytokinesis-blocked micronucleus (CBMN) assay

The cytokinesis-block micronucleus assay (CBMN), performed according to the protocol described by M. Fenech [31, 32] and to the recent recommendations concerning exposure to nanomaterials [56–58], allowed us to identify chromosome breakage or loss following exposure to poorly soluble Co_3_O_4_P and cobalt chloride.

BEAS-2B cells were seeded onto a 2-well Lab-Tek™ II Chamber Slide™ System (Nalgene Nunc International, Villebon sur Yvette, France) at a density of 2 x 10^4^ cells cm^-2^. After 24 h culture, cells were treated with increasing concentrations of Co_3_O_4_P and/or CoCl_2_ (0 to 100 μg mL^-1^ cobalt), with 0.1 μg mL^-1^ mitomycin C (MMC), which served as a positive control, whereas LHC-9 culture medium and LB-3 (50 μg mL^-1^) were the negative controls. After 24 h exposure, cells were washed and cytochalasin B (3 μg mL^-1^) was added to the cultures to block cytokinesis. Cells were maintained in culture for an additional 28 h in order to arrest the cytokinesis and to allow the scoring of binucleated cells. Afterwards, cells were fixed with 4 % paraformaldehyde in PBS (20 min, RT) and permeabilized with 0.25 % Triton® X-100. To stain the cytoplasm, actin staining was carried out with a solution of phalloidin-tetramethylrhodamine B isothiocyanate (0.06 μg mL^-1^), while the nuclear staining was performed using ProLong® Gold antifade reagent with DAPI.

All assays were performed in two independent experiments, and slides were scored blindly using an epifluorescence microscope (Nikon; Champigny sur Marne, France) at 400 X magnification.

Micronuclei (MN) were assessed in binucleated (BN) cells that had completed one nuclear division following exposure to the test compounds [59–61]. As previously described [53], for each experimental condition the number of BN micronucleated (BNMN) cells was scored in 1000 BN cells.

Since CBMN was performed in its implemented cytome version [32], cytostasis and cytotoxicity exerted by poorly soluble Co_3_O_4_P and cobalt chloride in BEAS-2B cells were also determined. To evaluate cytostasis, the cytokinesis block proliferation index (CBPI) was calculated by scoring mononucleated, BN and multinucleated cells in the first 500 living cells analyzed. CBPI, which indicates the average number of cell divisions completed by the cells, was calculated as follows [61]: [(1 x number of mononucleated cells) + (2 x number of BN cells) + (3 x number of multinucleated cells)/500 cells]. The percentage of cytostasis was calculated as described by OECD (TG487, 2014): {100 - 100 x [(CBPI treated cells - 1)/(CBPI C neg - 1)]}.

Finally, the apoptotic index, calculated as the percentage of apoptotic cells (early and late apoptosis) in 500 viable cells [32], allowed us to evaluate the cytotoxic potential of cobalt in bronchial BEAS-2B cells.

### Alkaline comet assay: primary and oxidative DNA damage

To detect the DNA damage induced by cobalt in BEAS-2B cells, the comet assay in alkaline conditions was performed. 2.0 x 10^4^ cells cm^-2^ were seeded onto precoated 12-well plates (BD Falcon; Le Pont de Claix, France) and, after overnight incubation, cultures were exposed to poorly soluble Co_3_O_4_P, CoCl_2_ and LB-3 for 2 and 24 h. At the end of the treatment period, cells were trypsinized, diluted in 1 % low-melting-point (LMP) agarose and spotted onto glass slides precoated with 1.6 % and 0.8 % normal-melting-point (NMP) agarose. Afterwards, cells were lysed and the DNA denatured. After denaturation, which occurred in a solution comprising 300 mM NaOH and 1 mM EDTA in MilliQ water, slides underwent electrophoresis by setting constant voltage (25 V) and variable amperage (300 to 315 mA) for 20 min at 4 °C. After neutralization and dehydration, slides were air-dried before being stained with propidium iodide (PI). As a negative control, cells were incubated in LHC9 medium alone, while as a positive control cells were exposed (5 min at 4 °C, protected from light) to 110 μM hydrogen peroxide (H_2_O_2_).

For the analysis of oxidative DNA damage, after cell membrane lysis, the enzymes, formamidopyrimidine DNA glycosylase (FPG; New England Biolabs, Evry, France) and human 8-oxoguanine DNA N-glycosylase 1 (hOGG1; New England Biolabs, Evry, France), were added to the slides and incubated for 30 min at 37 °C.

Slides, which were prepared in duplicate for each experimental condition, were analyzed under a fluorescence microscope at 400 X magnification using the Komet 6.0 software (Andor Bioimaging, Nottingham, UK). DNA damage was expressed as mean tail DNA % ± standard error of the mean (SEM).

### Immunostaining of gamma-H2Ax foci

The induction of DNA double strand breaks (DSB) following exposure to poorly soluble Co_3_O_4_P was analyzed by gamma-H2Ax immunostaining. To examine the role of ROS in the production of DSB after exposure to Co_3_O_4_P and cobalt chloride, we examined DNA damage in the presence/absence of pretreatment with the ROS scavenger, N-acetyl-cysteine (NAC). After seeding (2 x 10^4^ cells cm^-2^ onto 2-well Lab-Tek™ II Chamber Slide™ System), cells were exposed to a subtoxic concentration (0.5 mM) of NAC for 2 h before the addition of increasing concentrations of Co_3_O_4_P and CoCl_2_ (0–20 μg mL^-1^ cobalt). After 24 h treatment, cells were washed with PBS, fixed for 20 min in 4 % paraformaldehyde in PBS, and permeabilized for 2 min at 4 °C in a buffer composed of 20 mM Tris at pH 7, 20 mM NaCl, 300 mM sucrose, 3 mM MgCl_2_ and 0.5 % Triton X-100. Thereafter, BEAS-2B cells were incubated with the anti-gamma H2Ax ser139 antibody (clone JBW301; Merck Millipore, Fontenay sous Bois, France) for 40 min at 37 °C. After two washes in PBS, cells were then incubated (20 min, 37 °C) with an anti-mouse fluorescein (FITC) secondary antibody (Sigma-Aldrich, France). Nuclear staining was performed with DAPI Prolong® Gold antifade reagent. Slides were observed at 400 X magnification under a fluorescence microscope (Nikon; Champigny sur Marne, France). Experiments were performed in duplicate, and for each sample two slides were observed (500 cells per slide were scored and analyzed, or 200 cells for NAC experiments).

### Statistical analysis

Data are presented as the mean ± SEM. The Prism6 software (GraphPad software; La Jolla, CA, USA) allowed us to calculate IC_50_ values, to test if the effect was dose dependent and to analyze statistical significance (one-way ANOVA followed by Holm-Sidak method for multiple comparisons). CBMN statistical analysis was performed by chi-square test. Statistical significance was set as *p < 0.05, **p < 0.01 and ***p < 0.001.

## Abbreviations

8-oxoGua: 8-Oxoguanine
BN: binucleated cells
BNMN: binucleated micronucleated cells
CBMN: cytokinesis-block micronucleus assay
CBPI: cytokinesis-block proliferation index
Co_3_O_4_P: poorly soluble cobalt (II,III) oxide particles
CoCl_2_: soluble cobalt chloride
CoOP: cobalt (II) oxide particles
CoP: metallic cobalt particles
DLS: dynamic light scattering
DSB: double strand breaks
FPG: formamidopyrimidine DNA glycosylase
hOGG1: 8-oxoguanine DNA N-glycosylase 1
ICP-AES: inductively coupled plasma atomic emission spectroscopy
LB-3: polystyrene latex beads 0.3 μm mean particle size
MMC: mitomycin C
MN: micronuclei
NAC: N-acetylcysteine
ROS: reactive oxygen species
SSB: single strand breaks
TEM: transmission electron microscopy
γ-H2Ax: phosphorylated histone H2A

## References

[1] Mehrer SK, Rao GR (2011). Ultralayered Co3O4 for High-Performance Supercapacitor Applications. J Phys Chem C.

[2] Liu X, Long Q, Jiang C, Zhan B, Li C, Liu S (2013). Facile and green synthesis of mesoporous Co3O4 nanocubes and their applications for supercapacitors. Nanoscale.

[3] Miller JR, Simon P (2008). Materials science. Electrochemical capacitors for energy management. Science.

[4] Shim BS, Chen W, Doty C, Xu C, Kotov NA (2008). Smart electronic yarns and wearable fabrics for human biomonitoring made by carbon nanotube coating with polyelectrolytes. Nano Lett.

[5] Na CW, Woo HS, Kim ID, Lee JH (2011). Selective detection of NO2 and C2H5OH using a Co3O4-decorated ZnO nanowire network sensor. Chem Commun (Camb).

[6] Bekermann D, Gasparotto A, Barreca D, Maccato C, Comini E, Sada C (2012). Co3O4/ZnO nanocomposites: from plasma synthesis to gas sensing applications. ACS Appl Mater Interfaces.

[7] Bouchard LS, Anwar MS, Liu GL, Hann B, Xie ZH, Gray JW (2009). Picomolar sensitivity MRI and photoacoustic imaging of cobalt nanoparticles. Proc Natl Acad Sci U S A.

[8] Liu L, Zhang H, Meng X, Yin J, Li D, Liu C (2010). Dinuclear metal(II) complexes of polybenzimidazole ligands as carriers for DNA delivery. Biomaterials.

[9] Yin J, Meng X, Zhang S, Zhang D, Wang L, Liu C (2012). The effect of a nuclear localization sequence on transfection efficacy of genes delivered by cobalt (II)-polybenzimidazole complexes. Biomaterials.

[10] Jiang R, Yin J, Hu S, Meng X, Liu C (2013). Cobalt (II)-polybenzimidazole complexes as a nonviral gene carrier: effects of charges and benzimidazolyl groups. Curr Drug Deliv.

[11] Le Guen B, Ansoborlo E (2005). Le Cobalt et ses Isotopes.

[12] Davis K, Marsh JW, Gerondal M, Bailey MR, Le Guen B (2007). Assessment of intakes and doses to workers followed for 15 years after accidental inhalation of ^60^CO. Health Phys.

[13] Collier CG, Pearce MJ, Hodgson A, Ball A (1992). Factors affecting the in vitro dissolution of cobalt oxide. Environ Health Perspect.

[14] Barceloux DG (1999). Cobalt. J Toxicol Clin Toxicol.

[15] Papis E, Rossi F, Raspanti M, Dalle-Donne I, Colombo G, Milzani A (2009). Engineered cobalt oxide nanoparticles readily enter cells. Toxicol Lett.

[16] Sabbioni E, Ponti J, Del Torchio R, Farina M, Fortaner S, Munaro B (2006). Recherche in vitro sur la toxicologie des nanoparticules au Joint Research Center. Med Nucl Imag Funct Metabol.

[17] Ortega R, Bresson C, Darolles C, Gautier C, Roudeau S, Perrin L (2014). Low-solubility particles and a Trojan-horse type mechanism of toxicity: the case of cobalt oxide on human lung cells. Part Fibre Toxicol.

[18] Sabbioni E, Fortaner S, Farina M, Del Torchio R, Olivato I, Petrarca C (2014). Cytotoxicity and morphological transforming potential of cobalt nanoparticles, microparticles and ions in Balb/3 T3 mouse fibroblasts: an in vitro model. Nanotoxicology.

[19] Smith LJ, Holmes AL, Kandpal SK, Mason MD, Zheng T, Wise JPS (2014). The cytotoxicity and genotoxicity of soluble and particulate cobalt in human lung fibroblast cells. Toxicol Appl Pharmacol.

[20] Gault N, Sandre C, Poncy JL, Moulin C, Lefaix JL, Bresson C (2010). Cobalt toxicity: chemical and radiological combined effects on HaCaT keratinocyte cell line. Toxicol In Vitro.

[21] Kühnel D, Scheffler K, Wellner P, Meißner T, Potthoff A, Busch W (2012). Comparative evaluation of particle properties, formation of reactive oxygen species and genotoxic potential of tungsten carbide based nanoparticles in vitro. J Hazard Mater.

[22] Ponti J, Sabbioni E, Munaro B, Broggi F, Marmorato P, Franchini F (2009). Genotoxicity and morphological transformation induced by cobalt nanoparticles and cobalt chloride: an in vitro study in Balb/3 T3 mouse fibroblasts. Mutagenesis.

[23] Li Q, Ke Q, Costa M (2009). Alterations of histone modifications by cobalt compounds. Carcinogenesis.

[24] Horev-Azaria L, Kirkpatrick CJ, Korenstein R, Marche PN, Maimon O, Ponti J (2011). Predictive toxicology of cobalt nanoparticles and ions: comparative in vitro study of different cellular models using methods of knowledge discovery from data. Toxicol Sci.

[25] Forbes II (2000). Human airway epithelial cell lines for in vitro drug transport and metabolism studies. Pharm Sci Technolo Today.

[26] Courcot E, Leclerc J, Lafitte JJ, Mensier E, Jaillard S, Gosset P (2012). Xenobiotic metabolism and disposition in human lung cell models: comparison with in vivo expression profiles. Drug Metab Dispos.

[27] Malard V, Berenguer F, Prat O, Ruat S, Steinmetz G, Quemeneur E (2012). Global gene expression profiling in human lung cells exposed to cobalt. BMC Genomics.

[28] Bresson C, Darolles C, Carmona A, Gautier C, Sage N, Roudeau S (2013). Cobalt chloride speciation, mechanisms of cytotoxicity on human pulmonary cells, and synergistic toxicity with zinc. Metallomics.

[29] Darolles C, Sage N, Armengaud J, Malard V (2013). In vitro assessment of cobalt oxide particle toxicity: identifying and circumventing interference. Toxicol In Vitro.

[30] Cavallo D, Ciervo A, Fresegna AM, Maiello R, Tassone P, Buresti G (2015). Investigation on cobalt-oxide nanoparticles cyto-genotoxicity and inflammatory response in two types of respiratory cells. J Appl Toxicol.

[31] Fenech M (2006). Cytokinesis-block micronucleus assay evolves into a “cytome” assay of chromosomal instability, mitotic dysfunction and cell death. Mutat Res.

[32] Fenech M (2007). Cytokinesis-block micronucleus cytome assay. Nat Protoc.

[33] Calzolai L, Gilliland D, Garcìa CP, Rossi F (2011). Separation and characterization of gold nanoparticle mixtures by flow-field-flow fractionation. J Chromatogr A.

[34] Berne B, Pecora R (2000). Dynamic Light Scattering with Applications to Chemistry, Biology, and Physics.

[35] OECD: Test No. 487: In Vitro Mammalian Cell Micronucleus Test, OECD Guidelines for the Testing of Chemicals, Section 4, OECD Publishing, Paris. 2014, doi: http://dx.doi.org/10.1787/9789264224438-en.

[36] Smith CC, O’Donovan MR, Martin EA (2006). hOGG1 recognizes oxidative damage using the comet assay with greater specificity than FPG or ENDOIII. Mutagenesis.

[37] Christensen JM, Poulsen OM (1994). A 1982-1992 surveillance programme on Danish pottery painters. Biological levels and health effects following exposure to soluble or insoluble cobalt compounds in cobalt blue dyes. Sci Total Environ.

[38] Lundborg M, Falk R, Johansson A, Kreyling W, Camner P (1992). Phagolysosomal pH and dissolution of cobalt oxide particles by alveolar macrophages. Environ Health Perspect.

[39] Limbach LK, Wick P, Manser P, Grass RN, Bruinink A, Stark WJ (2007). Exposure of engineered nanoparticles to human lung epithelial cells: influence of chemical composition and catalytic activity on oxidative stress. Environ Sci Technol.

[40] IARC monographs on the evaluation of carcinogenic risks to humans: Cobalt in Hard Metals and Cobalt Sulfate, Gallium Arsenide, Indium Phosphide and Vanadium Pentoxide. IARC Monographs. 2006;86. PMID:16906675. [PubMed - indexed for MEDLINE]. http://monographs.iarc.fr/ENG/Monographs/vol86/mono86.pdf. PMC4781610 16906675

[41] Horie M, Fujita K, Kato H, Endoh S, Nishio K, Komaba LK (2012). Association of the physical and chemical properties and the cytotoxicity of metal oxide nanoparticles: metal ion release, adsorption ability and specific surface area. Metallomics.

[42] Chattopadhyay S, Dash SK, Kar Mahapatra S, Tripathy S, Ghosh T, Das B (2014). Chitosan-modified cobalt oxide nanoparticles stimulate TNF-α-mediated apoptosis in human leukemic cells. J Biol Inorg Chem.

[43] Alarifi S, Ali D, AO Y, Ahamed M, Siddiqui MA, Al-Khedhairy AA (2013). Oxidative stress contributes to cobalt oxide nanoparticles-induced cytotoxicity and DNA damage in human hepatocarcinoma cells. Int J Nanomedicine.

[44] Sabbioni E, Fortaner S, Farina M, Del Torchio R, Petrarca C, Bernardini G (2014). Interaction with culture medium components, cellular uptake and intracellular distribution of cobalt nanoparticles, microparticles and ions in Balb/3 T3 mouse fibroblasts. Nanotoxicology.

[45] Colognato R, Bonelli A, Ponti J, Farina M, Bergamaschi E, Sabbioni E (2008). Comparative genotoxicity of cobalt nanoparticles and ions on human peripheral leukocytes in vitro. Mutagenesis.

[46] Annangi B, Bach J, Vales G, Rubio L, Marcos R, Hernández A (2015). Long-term exposures to low doses of cobalt nanoparticles induce cell transformation enhanced by oxidative damage. Nanotoxicology.

[47] Wan R, Mo Y, Feng L, Chien S, Tollerud DJ, Zhang Q (2012). DNA damage caused by metal nanoparticles: involvement of oxidative stress and activation of ATM. Chem Res Toxicol.

[48] De Boeck M, Lombaert N, De Backer S, Finsy R, Lison D, Kirsch-Volders M (2003). In vitro genotoxic effects of different combinations of cobalt and metallic carbide particles. Mutagenesis.

[49] AshaRani PV, Low Kah Mun G, Hande MP, Valiyaveettil S (2009). Cytotoxicity and genotoxicity of silver nanoparticles in human cells. ACS Nano.

[50] Kermanizadeh A, Gaiser BK, Hutchison GR, Stone V (2012). An in vitro liver model--assessing oxidative stress and genotoxicity following exposure of hepatocytes to a panel of engineered nanomaterials. Part Fibre Toxicol.

[51] Magdolenova Z, Collins A, Kumar A, Dhawan A, Stone V, Dusinska M (2014). Mechanisms of genotoxicity. A review of in vitro and in vivo studies with engineered nanoparticles. Nanotoxicology.

[52] Chattopadhyay S, Dash SK, Tripathy S, Das B, Mandal D, Pramanik P (2015). Toxicity of cobalt oxide nanoparticles to normal cells; an in vitro and in vivo study. Chem Biol Interact.

[53] Chattopadhyay S, Dash SK, Tripathy S, Das B, Kar Mahapatra S, Pramanik P (2015). Cobalt oxide nanoparticles induced oxidative stress linked to activation of TNF-α/caspase-8/p38-MAPK signaling in human leukemia cells. J Appl Toxicol.

[54] Alinovi R, Goldoni M, Pinelli S, Campanini M, Aliatis I, Bersani D (2015). Oxidative and pro-inflammatory effects of cobalt and titanium oxide nanoparticles on aortic and venous endothelial cells. Toxicol In Vitro.

[55] Ayala A, Muñoz MF, Argüelles S (2014). Lipid peroxidation: production, metabolism, and signaling mechanisms of malondialdehyde and 4-hydroxy-2-nonenal. Oxid Med Cell Longev.

[56] Gonzalez L, Decordier I, Kirsch-Volders M (2010). Induction of chromosome malsegregation by nanomaterials. Biochem Soc Trans.

[57] Gonzalez L, Sanderson BJ, Kirsch-Volders M (2011). Adaptations of the in vitro MN assay for the genotoxicity assessment of nanomaterials. Mutagenesis.

[58] Gonzalez L, Corradi S, Thomassen LC, Martens JA, Cundari E, Lison D (2011). Methodological approaches influencing cellular uptake and cyto-(geno) toxic effects of nanoparticles. J Biomed Nanotechnol.

[59] Fenech M (2000). The in vitro micronucleus technique. Mutat Res.

[60] Kirsch-Volders M, Sofuni T, Aardema M, Albertini S, Eastmond D, Fenech M (2010). Report from the In Vitro Micronucleus Assay Working Group. Environ Mol Mutagen.

[61] Kirsch-Volders M, Sofuni T, Aardema M, Albertini S, Eastmond D, Fenech M (2003). Report from the in vitro micronucleus assay working group. Mutat Res.

